# Prediction of Stress–Strain Curves for HFRP Composite Confined Brick Aggregate Concrete under Axial Load

**DOI:** 10.3390/polym15040844

**Published:** 2023-02-08

**Authors:** Panumas Saingam, Ali Ejaz, Nazam Ali, Adnan Nawaz, Qudeer Hussain, Panuwat Joyklad

**Affiliations:** 1 Department of Civil Engineering, School of Engineering, King Mongkut’s Institute of Technology Ladkrabang, Bangkok 10520, Thailand; 2 National Institute of Transportation, National University of Sciences and Technology (NUST), Islamabad 44000, Pakistan; 3 Department of Civil Engineering, COMSATS University Islamabad, Sahiwal Campus, Sahiwal 57000, Pakistan; 4 Department of Civil Engineering, COMSATS University Islamabad, Wah Campus, Wah 47040, Pakistan; 5 Center of Excellence in Earthquake Engineering and Vibration, Department of Civil Engineering, Chulalongkorn University, Bangkok 10330, Thailand; 6 Department of Civil and Environmental Engineering, Faculty of Engineering, Srinakharinwirot University, Nakhonnayok 26120, Thailand

**Keywords:** fired-clay brick aggregates: recycled aggregate concrete, hemp fiber rope, stress–strain models

## Abstract

Recently, hemp-fiber-reinforced polymer (HFRP) composites have been developed to enhance the strength and ductility of normal and lightweight aggregate concrete along with recycled brick aggregate concrete. In addition, both experimental and analytical investigations have been performed to assess the suitability of the existing strength and strain models. However, the theoretical and analytical expressions to predict the stress–strain curves of HFRP-confined concrete were not developed. Therefore, the main objective of this study was to develop analytical expressions to predict the stress–strain curves of HFRP-confined waste brick aggregate concrete. For this purpose, a new experimental framework was conducted to examine the effectiveness of HFRP in improving the mechanical properties of concrete constructed with recycled brick aggregates. Depending on the strength of the concrete, two groups were formed, i.e., Type-1 concrete and Type-2 concrete. A total of sixteen samples were tested. The ultimate compressive strength and strain significantly increased due to HFRP confinement. Improvements of up to 272% and 457% in the ultimate compressive strength and strain were observed due to hemp confinement, respectively. To predict the ultimate compressive strength and strain of HFRP-confined concrete, this study investigated several existing analytical stress–strain models. Some of the strength models resulted in close agreement with experimental results, but none of the models could accurately predict the ultimate confined strain. Nonlinear regression analysis was conducted to propose expressions to predict the ultimate compressive strength and strain of HFRP-confined concrete. The proposed expressions resulted in good agreement with experimental results. An analytical procedure was proposed to predict the stress–strain curves of hemp-confined concrete constructed by partial replacement of natural coarse aggregates by recycled fired-clay brick aggregates. A close agreement was found between the experimental and analytically predicted stress–strain curves.

## 1. Introduction

A significant number of nonrenewable resources have been used in the manufacturing of concrete and mortar, which involves adverse environmental effects. Some nonrenewable resources such as sand, gravel, and cement combine to form concrete. Approximately 60% of raw materials from the lithosphere are used in construction globally. Additionally, extensive construction activities have resulted in the depletion of natural resources [1]. Importing aggregates to regions that lack high-quality rocks or gravel is not economical. Besides this, good natural aggregates are difficult to locate, and mining has become more difficult, whereas sand and stone supplies have been increasingly depleted in many urban areas.

Environmental protection, the prevention of nonrenewable resources, and energy conservation all work together in environment-friendly development processes [2,3,4]. Issues related to bad design, poor construction, and inferior materials have forced numerous buildings to reach their end of life prematurely or develop durability-related issues. Furthermore, a lot of waste is generated by periodic earthquakes damaging substandard buildings [5,6]. Worldwide, construction using fired-clay brick is very common [7]. Bricks are a common material in construction and have long been a crucial component of residential and other low-rise buildings. Moreover, the construction of building boundary walls frequently utilizes clay bricks. According to reports, China produces about 15.5 million tons of construction waste each year, a significant proportion of which comprises concrete and bricks [3]. According to another survey conducted by the European Union in 2011, the continent produces about 1 billion tons of construction waste each year, which also involves bricks in significant quantity [8]. The potential use of crushed fired-clay bricks as a novel civil engineering material needs to be investigated to overcome the high cost of natural aggregates and the scarcity of their natural resources. In addition to lowering the expense of site cleanup and disposal, the use of leftover fired-clay bricks has positive social and economic effects on the environment [9]. The use of waste brick aggregates as a partial replacement for natural aggregates in concrete has been investigated in the past. Several related research works are summarized below.

According to Yang et al. [10], a 20% and 50% replacement of the natural aggregates in concrete with fired-clay brick aggregates reduced the compressive strength by 11% and 20%, respectively. The mechanical properties of recycled aggregate concrete (RAC) are greatly influenced by the potential of recycled aggregates to retain more water than natural aggregates. In another study conducted by Novakova and Mikulica [11], the particle density of RAC was found to be five to fifteen times lower than that of natural aggregate concrete. This was attributed to the lower density of recycled aggregates resulting from the surface-adhered mortar. González et al. [12] revealed that the compressive strength could be reduced by a maximum of 28% when recycled brick aggregates replaced 100% of the natural aggregates. Further, up to a replacement ratio of 35%, no significant effect on compressive strength was observed. These findings imply that the driving forces behind the inferior attributes of recycled brick aggregate concrete include their increased water absorption, lower density, and lower resistance to fragmentation as compared to natural aggregates. In another study [13], it was found that for structural applications, an upper limit of 30% on the replacement ratio of natural aggregates must be imposed to maintain the appropriate strength of concrete. Debieb and Kenai [14] showed that the compressive strength of RAC was reduced by 30% when recycled aggregates replaced 100% of the natural aggregates. The compressive strength decreased by 39% when 40% of the natural aggregates were substituted with recycled aggregates [15]. Cachim [16] concluded that a replacement ratio of up to 15% had no impact on concrete properties, whereas a 20% reduction in the properties of concrete was observed for a 30% replacement ratio. Liu et al. [17] suggested that cracks propagate through brick materials in recycled brick aggregate concrete, which is the primary cause of the low strength of the resulting concrete. Despite possessing inferior attributes, recycled brick aggregates have certain benefits, including their light weight having enhanced fire resistance attributed to the refractory properties of bricks [18].

Concrete made using recycled brick aggregates has inferior characteristics to natural aggregate concrete [19,20,21,22]. When recycled brick aggregates replace natural aggregates at a replacement ratio lower than 30%, the reduction in mechanical characteristics has been found to be negligible. Thus, the goal of the current study is to improve the mechanical properties of recycled fired-clay brick aggregate concrete at replacement ratios of more than 30%. Utilizing various natural and synthetic fiber-reinforced polymer (FRP) sheets to wrap RAC is a common technique to enhance its mechanical properties. Synthetic FRPs are widely employed nowadays in structural strengthening projects [16,23,24,25,26] and to improve the structural properties of RAC [27,28,29,30]. However, the high cost of synthetic FRPs is a major concern [31]. Additionally, the chemicals used in the production of synthetic FRPs have the potential to cause skin problems such as allergies and irritating contact dermatitis in susceptible persons. Natural fibers have recently been suggested as a potential replacement for synthetic FRPs [32]. Compared to synthetic FRPs, natural FRPs are significantly cheaper and do not pose the threat of skin infections [33,34].

Cotton, jute, flax, sisal, and hemp are some eco-friendly natural fibers made from textile and agricultural production that have been effectively employed for concrete repair and reinforcement [35,36]. These fibers are affordable and produce very little CO_2_ during production. The impact of jute, sisal, and hemp fibers on the strain and the ultimate compressive strength of concrete was examined by Hussain et al. [37]. It was found that specimens strengthened by hemp fiber ropes demonstrated the highest increase in ultimate compressive strength. In another study by Fragoudakis et al. [38], it was shown that hemp ropes significantly improved the deflection of concrete beams and bending strain. Three layers of hemp ropes were used by Ghalieh et al. [39] to wrap concrete columns. Different column slenderness ratios were considered. In comparison to their respective reference columns, confined columns exhibited higher axial strength and ductility.

**Figure 1:**
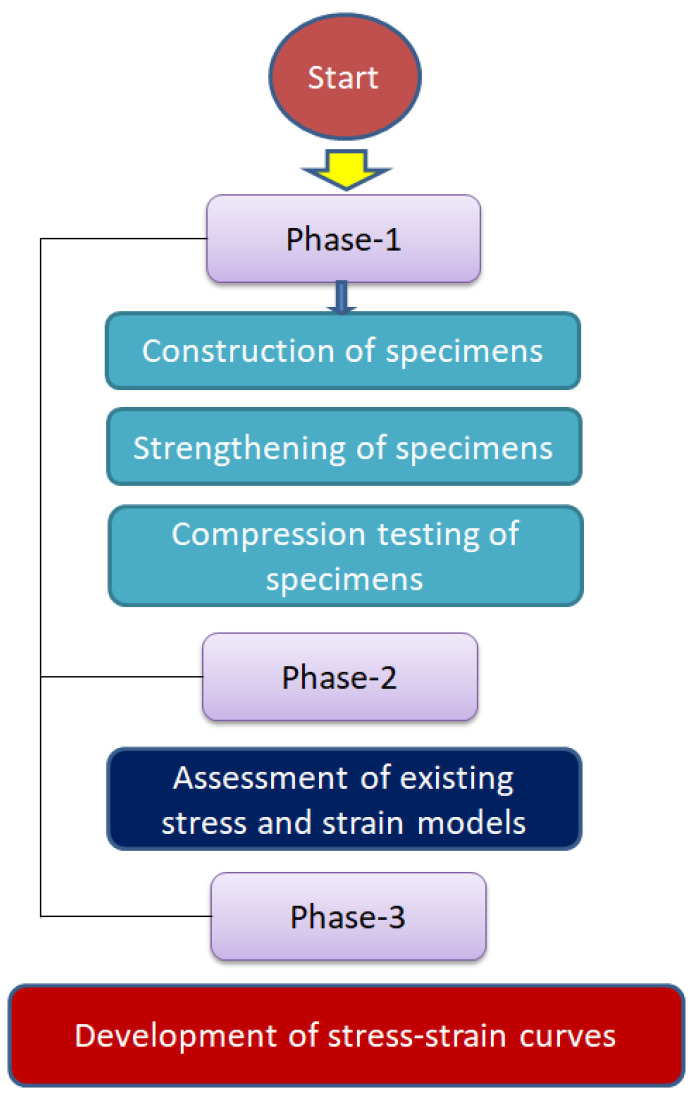
Research flow chart.

The goal of this research work is to enhance the subpar mechanical characteristics of recycled aggregate concrete (RAC) that contains a 50% replacement of natural aggregates with fired-clay brick aggregates (CBB). Recently, a study was conducted to partially replace natural aggregates with hydraulically pressed cement brick aggregates [40]. However, the current study investigates the effectiveness of fired-clay brick aggregate waste by replacing 50% of natural aggregates in concrete. To date, no study has been conducted to assess the performance of hemp confinement in improving the mechanical properties of concrete that contain recycled fired-clay brick aggregates. In addition, this is a novel work to propose theoretical expressions for predicting the complete compressive stress–strain response of hemp-confined recycled brick aggregate concrete. For the strengthening purpose, hemp fiber ropes were utilized due to their inexpensive, simple-to-use, and eco-friendly nature. The accuracy of existing compressive strength and strain models was investigated in predicting the compressive strength and strain of HFRP-confined concrete. At present, no research work has been conducted to predict the stress vs. strain curves of HFRP-confined concrete. The current study aims to fill this gap by proposing equations to define the complete stress vs. strain curves of HFRP-confined concrete. A systematic flowchart of the overall process is presented in Figure 1.

## 2. Experimental Setup

### 2.1. Test Matrix

A total of sixteen concrete cylinders were tested in two groups. The diameter and height of each cylinder were 150 mm and 300 mm, respectively. Each group comprised eight cylinders, and the cylinders in two groups were differentiated based on their concrete strength. In each group, two cylinders were tested without strengthening and served as control specimens. Hemp fiber rope layers were applied to the remaining six cylinders. One, two, and three layers of hemp fiber ropes were applied to assess the effect of the quantity of strengthening material on mechanical properties. In the present work, the average results of two cylinders from the control, one-layer strengthening, two-layer strengthening, and three-layer strengthening are reported. The attributes of the test matrix are illustrated in Table 1. The test samples were given names that represented their geometrical shapes, i.e., (“C” for circular) CBB was used for fired-clay brick aggregate waste in an external confinement configuration (i.e., 1H, 2H, 3H, and CON for the 1, 2, and 3 layers of hemp ropes, and control specimen), and LS and HS were used for Type-1 and Type-2 concrete, respectively.

### 2.2. Properties of Materials

**Figure 2:**
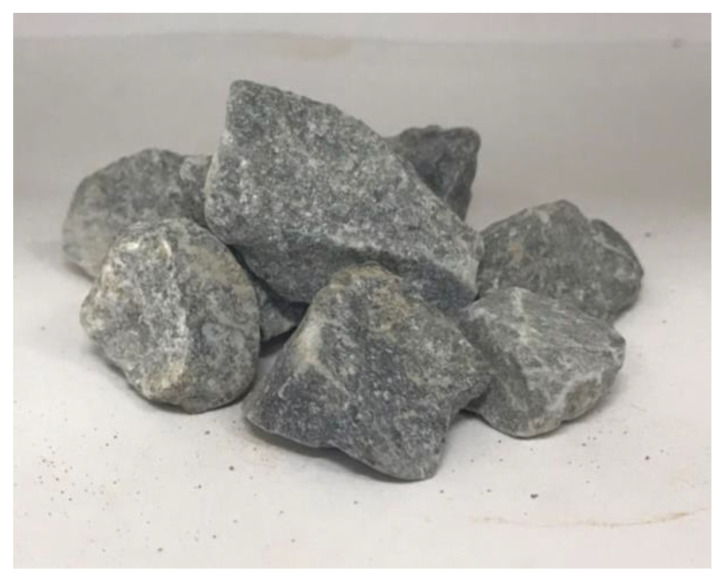
Natural aggregates.

**Figure 3:**
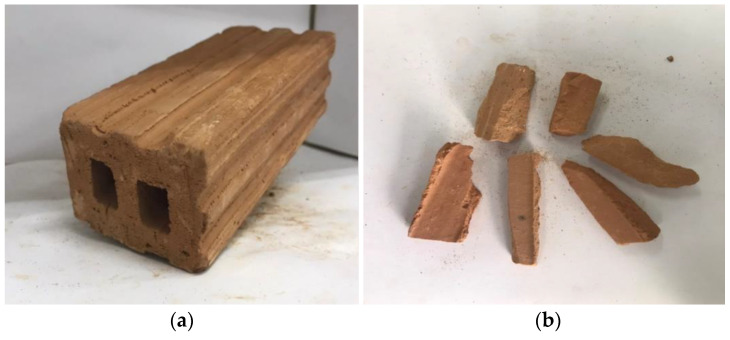
(**a**) Fired-clay interlocking bricks and (**b**) aggregate obtained from fired-clay interlocking bricks.

Type-I Portland cement was utilized for the construction of all cylinders. The selected slump values for Type-1 and Type-2 concrete were 90 mm and 70 mm, respectively. Fired-clay interlocking bricks made of clay were used to partially replace 50% of coarse natural aggregates. Natural aggregates are shown in Figure 2. Figure 3a shows fired-clay interlocking brick, whereas Figure 3 represents brick crushed aggregates with a maximum size of 25 mm.

ASTM standards were followed to estimate the mechanical properties of bricks, including their density, water absorption, and compressive strength [41,42]. The density of bricks was found at 145 (kg/m^3^), compressive strength at 6.26 (MPa), and water absorption at 12.30%. At the same time, the density of natural aggregates was approximately 2400–2600 kg/m^3^. Table 2 represents the mix proportions for Type-1 and Type-2 concrete.

**Figure 4:**
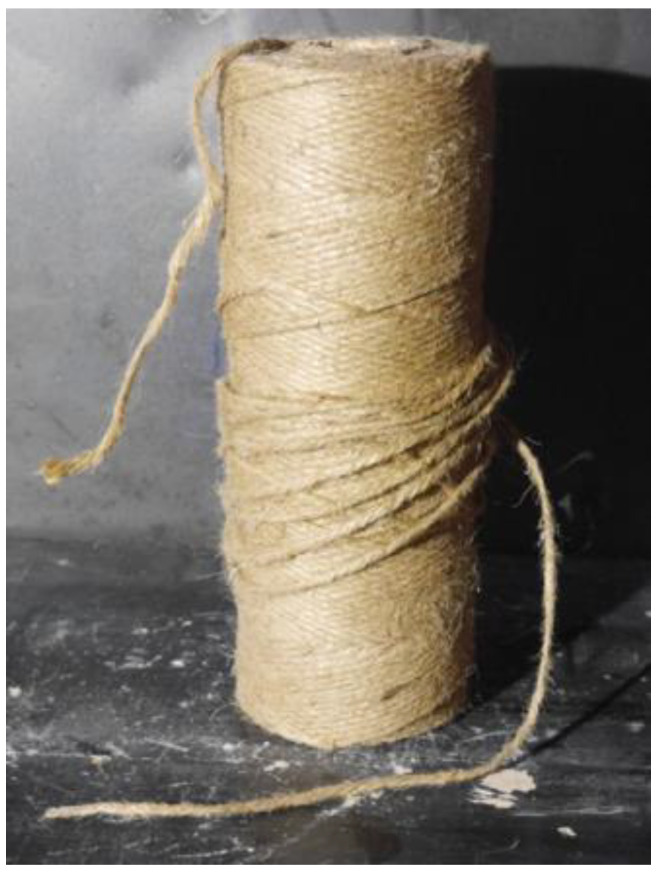
Hemp rope.

The hemp ropes were bonded to the cylinders by using a two-part polyester resin. The locally available polyester resin was used in this study. The polyester resin was obtained from RUGSVANICH (1992) company limited, Bangkok, Thailand. The mixing ratio of resin and hardener was 2:1. The produced epoxy was used to bond hemp ropes and concrete by using a hand brush. Table 3 illustrates the physical properties of epoxy resin. According to ASTM A931-18 [43] and ASTM E8/E8M-13 [44], the tensile properties of hemp fiber ropes were estimated. A loading rate of 1.5 mm/min was used to test the rope specimens. Figure 4 shows the hemp rope used in the present study. The diameter of the hemp rope was 2.1 mm. The estimated tensile strength and strain of hemp ropes were 137.4 MPa and 3.5%, respectively.

### 2.3. Strengthening Process

**Figure 5:**
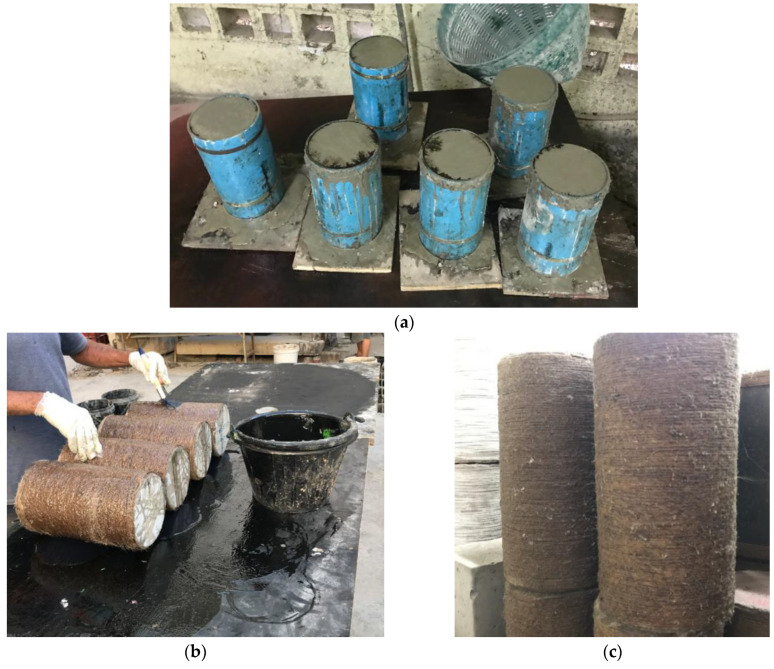
(**a**) Construction of specimens, (**b**) application of two-part epoxy and hemp ropes to cylindrical specimens, and (**c**) HFRP-confined specimens.

The strengthening of cylinders was performed after the curing period of 28 days. Hussain et al. [37] recognized that a pre-tension force of 10 MPa on hemp fiber ropes was necessary before they could apply the confining pressure. Hence, the hemp ropes were pre-tensioned at 10 MPa before their application. To manage this, a specialized mechanical system was developed such as the system adopted by Hussain et al. [37]. The surface of the epoxy was impregnated with epoxy. Initially, with the help of super glue, one end of the hemp rope was attached to the concrete surface. Following that, hemp fiber ropes were wrapped around cylinders along their height. After wrapping the full height, the other end of the hemp ropes was glued to the concrete surface. Epoxy was again applied to the attached hemp ropes to fully saturate them. A twelve-hour gap was provided before applying the second hemp fiber rope layer, and a similar procedure as that for the first layer was followed. Figure 5 shows the application process of hemp ropes using epoxy.

### 2.4. Instrumentation and Loading Setup

**Figure 6:**
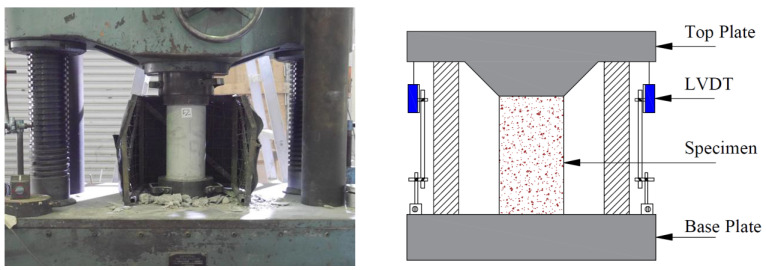
Mechanical testing setup.

To record the axial deformations, two linear variable displacement transducers (LVDTs) were fixed around cylinders. Each sample was subjected to a monotonic compressive load by utilizing a Universal Testing Machine with a 2 MN capacity. A loading rate of 4000 N/s was applied throughout the testing. To prevent any unintentional weight transfer to the fiber ropes at significant axial deformations, steel plates were mounted to the top and bottom sides of cylinders. A calibrated load cell was positioned at the top of the cylinders to measure the intensity of the load applied to it. The testing setup can be seen in Figure 6.

## 3. Experimental Results

### 3.1. Axial Stress–Strain Response

The experimental results in terms of compressive strength and strain and their improvements due to hemp fiber ropes are presented in Table 4. The application of one, two, and three layers of hemp fiber ropes increased the compressive strength in Group A by 88%, 187%, and 272%, respectively. The corresponding increase in Group B was identified at 52%, 99%, and 146%, respectively. Similarly, a 258%, 337%, and 457% increase in ultimate compressive strain was observed in Group A for one, two, and three layers of hemp fiber ropes, respectively. The corresponding increase in Group B was 51%, 262%, and 350%.

**Figure 7:**
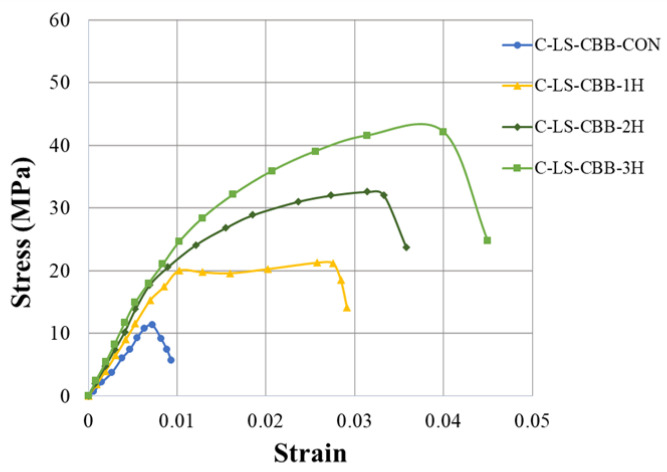
Stress–strain response of Type-1 concrete with and without hemp ropes confinement.

**Figure 8:**
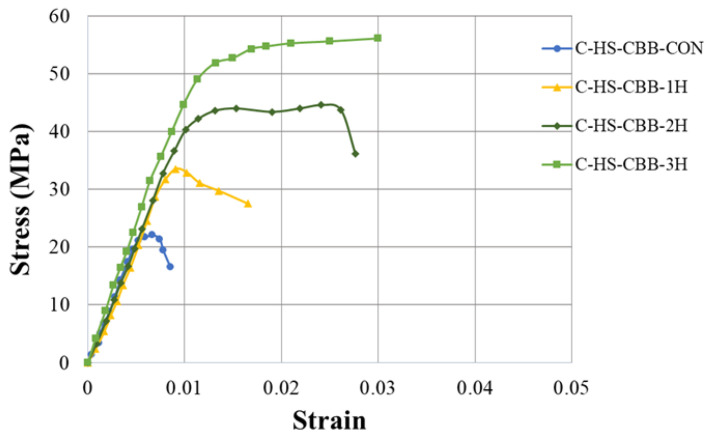
Stress–strain response of Type-2 concrete with and without hemp ropes confinement.

The compressive stress–strain curves of the control and hemp-confined specimens of the Type-1 and Type-2 strength concrete groups are shown in Figure 7 and Figure 8, respectively. It is clear that in both Type-1 and Type-2 concrete groups, the control specimens at their maximum compressive stresses showed brittle failure, as seen by a sharp decline in their axial strength. At the same time, the axial stress–strain response was bilinear for all HFRP-confined specimens. A similar bilinear curve was also observed by Jiang et al. [13] in their research work on recycled brick aggregate concrete confined by FRP. Figure 7 further demonstrates that as the number of hemp rope layers increased, the ultimate strength and the associated strain increased. However, for the hemp-rope-confined specimens, the second branch of the axial stress–strain curve appeared at almost similar axial strain values. Additionally, it was unable to distinguish between the initial slopes of confined samples. It can be inferred that regardless of the number of hemp fiber layers, they were capable of providing concrete with significant ductility and strength.

### 3.2. Hemp Rope Layers Effect on Type-1 and Type-2 Concrete

**Figure 9:**
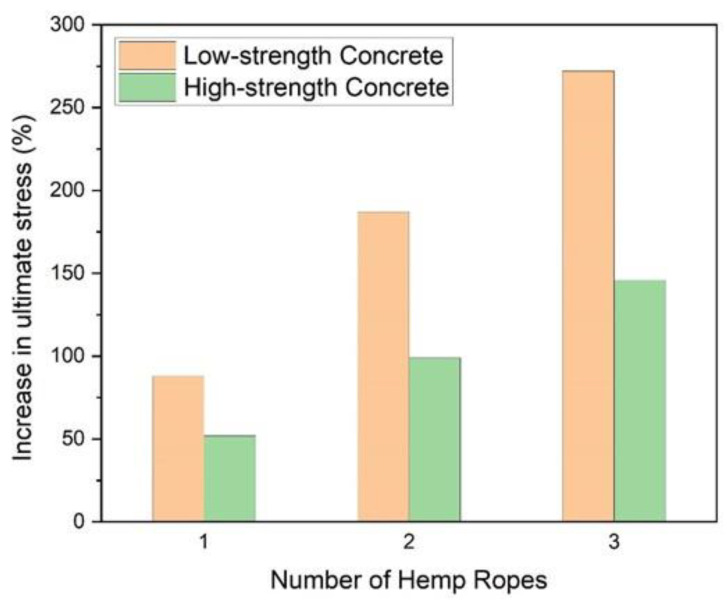
Comparison of increment in ultimate compressive strength due to number of hemp ropes (note: low-strength and high-strength concrete correspond to Type-1 and Type-2 concrete, respectively).

**Figure 10:**
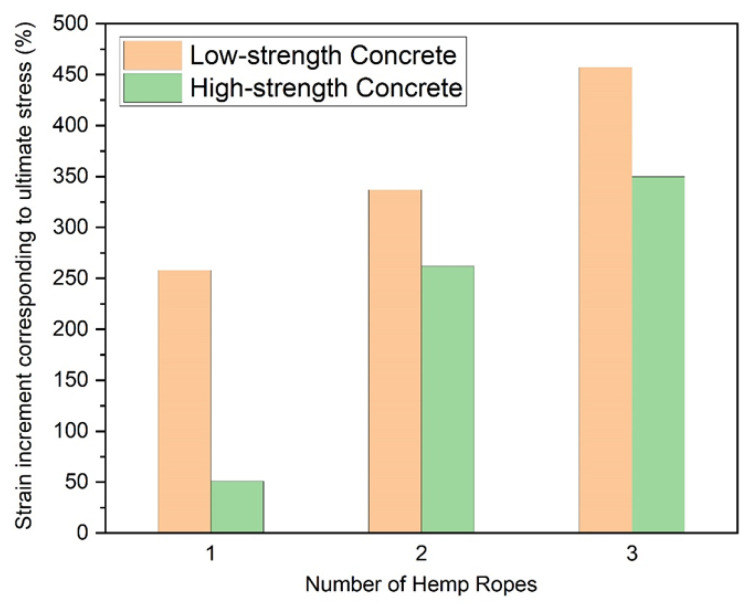
Comparison of increment in strain corresponding to ultimate compressive strength due to number of hemp ropes (note: low-strength and high-strength concrete correspond to Type-1 and Type-2 concrete, respectively).

The comparison of the increase in ultimate compressive strength and the corresponding strain of Type-1 and Type-2 concrete specimens confined by hemp ropes is shown in Figure 9 and Figure 10, respectively. For one, two, and three layers of hemp rope confinement, the ultimate strength of Type-1 concrete specimens was greater than that of Type-2 concrete specimens by 36%, 88%, and 126%, respectively. Similarly, the ultimate strain of Type-1 concrete specimens was greater than that of Type-2 concrete specimens by 207% for one, 75% for two, and 107% for three layers of hemp ropes. The performance of hemp rope confinement in Type-1 was better than that in Type-2 concrete. However, there is a positive association between the number of layers of hemp rope and the increase in ultimate strength and strain.

### 3.3. Failure Pattern of Specimens

**Figure 11:**
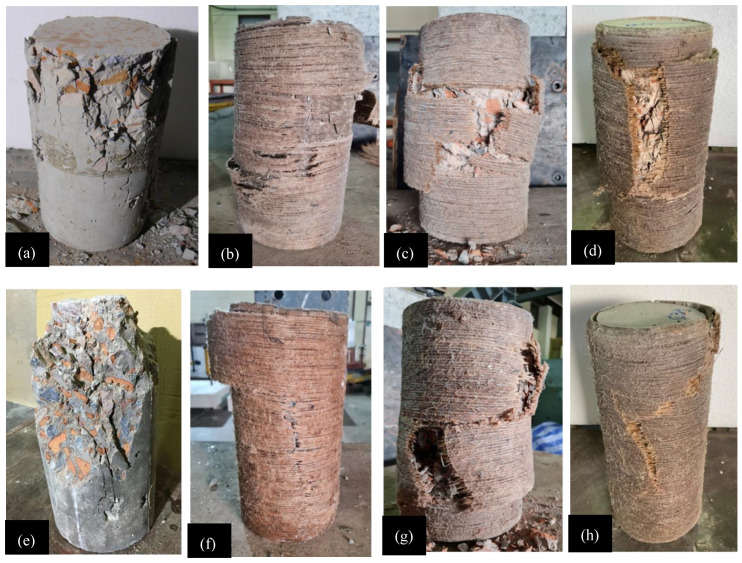
Ultimate failure pattern of specimens: (**a**) C-LS-CBB-CON, (**b**) C-LS-CBB-1H, (**c**) C-LS-CBB-2H, (**d**) C-LS-CBB-3H, (**e**) C-HS-CBB-CON, (**f**) C-HS-CBB-1H, (**g**) C-HS-CBB-2H, and (**h**) C-HS-CBB-3H.

Figure 11 shows the ultimate failure mode for each specimen. Crushing and splitting occurred along the height of the control specimens for both Type-1 and Type-2 concrete groups. Compared to the Type-1 concrete cylinder C-LS-CBB-CON, more crushing was found in Type-2 concrete cylinder C-HS-CBB-CON. Additionally, compared to the Type-1 control specimens, the Type-2 control specimens had a more abrupt failure pattern. Tensile failure of hemp ropes in the hoop direction caused all strengthened specimens to fail. As hemp ropes did not debond during the load application, it showed that the strength of epoxy was enough to maintain the bond between hemp ropes and the concrete surface throughout the loading. Snapping sounds were heard for samples reinforced with more than one layer of hemp rope, indicating the gradual fracturing of hemp ropes in underlying layers. This has been reported elsewhere [45]. The tensile fracture of hemp ropes occurred primarily within the central zone of samples confined with single and double layers. In contrast, the tensile fracture of the three-layer confinement spread over a wider area and up the entire height of the cylinders. This can be ascribed to the higher axial load and deformation sustained by the corresponding cylinders. Similar conclusions have also been reported in earlier studies [13,46].

## 4. Comparison with Analytical Models

### 4.1. Detail of Existing Models

Several researchers have proposed analytical models to predict the stress vs. strain behavior of concrete confined by Fiber-Reinforced Polymer (FRP) sheets. Some of the existing models are summarized in Table 5, including the models by Ghernouti and Rabehi, Legeron and Paultre, Triantafillou et al., Akiyama et al., and Benzaid et al. A consensus on the general form of the compressive strength of FRP-confined concrete exists as

(1) $$f_{cc}=\left[1+k_{1}\left(\frac{f_{l}}{f_{co}}\right)\right]f_{co}\;$$

where $f_{cc}$ and $f_{co}$ are the compressive strength of FRP-confined and unconfined concrete, respectively; $f_{l}$ represents the lateral confining pressure exerted by FRP sheets; $k_{1}$ represents the confinement effectiveness coefficient. The lateral confining pressure $f_{l}$ can be related to FRP thickness t and strength $f_{t}$ by using the formula shown below:

(2) $$fl=\frac{2f_{t}t}{D}$$

where $f_{t}$ denotes the tensile strength of FRP in the hoop direction, *t* describes the thickness of FRP sheets, and *D* defines the concrete core diameter.

### 4.2. Performance of Existing Models

**Figure 12:**
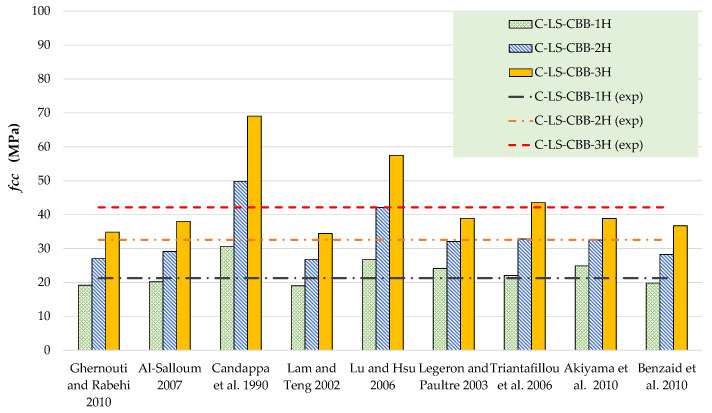
Comparison of strength for the Type-1 group [47,48,49,50,51,52,53,54,55].

**Figure 13:**
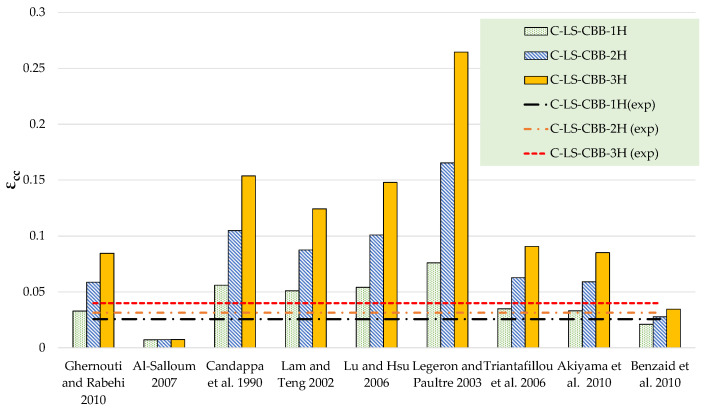
Comparison of strain for Type-1 group [47,48,49,50,51,52,53,54,55].

**Figure 14:**
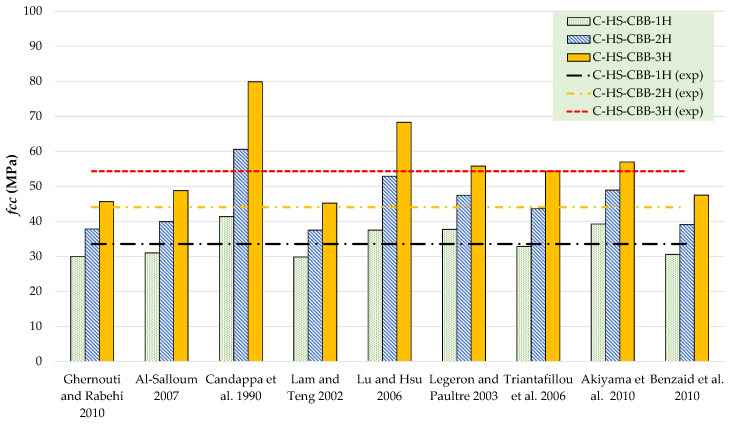
Comparison of strength for the Type-2 group [47,48,49,50,51,52,53,54,55].

**Figure 15:**
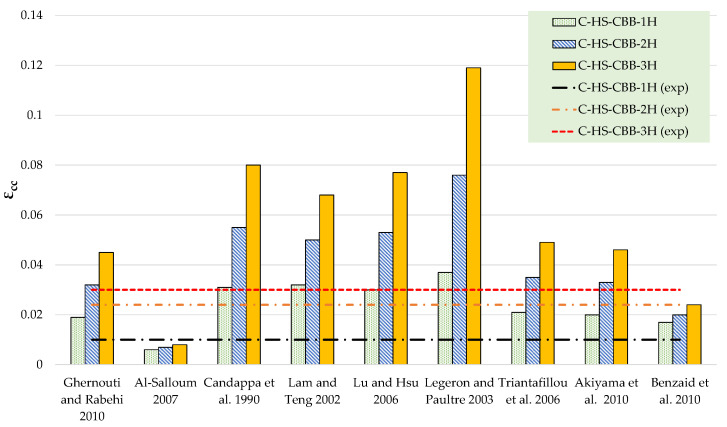
Comparison of strain for Type-2 group [47,48,49,50,51,52,53,54,55].

The results of theoretical compressive stress models are compared to the experimental results in Table 6 and Table 7 for Type-1 and Type-2 concrete specimens, respectively. Figure 12, Figure 13, Figure 14 and Figure 15 represent a graphical representation of the comparison of experimental and predicted compressive strength and strain values. It is evident that for a 2- and 3-layer hemp rope confinement, the predicted compressive strengths are higher for some models, whereas some models underestimate the experimental compressive strengths. However, the results of most strength models for confinement with 1 layer of hemp rope are closer to the experimental findings. For the Type-1 concrete group, the results of strength models given by Al-Salloum and Triantafillou et al. are closer to the experimental results of specimens having a single layer of CFRP confinement, whereas the models given by Legeron and Paultre, Triantafillou et al., and Akiyama et al. are close to the experimental results of specimens with 2-layer confinement. For theoretical predictions of Type-1 concrete having 3-layer confinement, only the model given by Triantafillou et al. is close to the experimental results. For the Type-2 concrete group, the results of strength models given by Al-Salloum, Triantafillou et al., and Benzaid et al. are close to the experimental results for 1-layer confinement, whereas the model given by Triantafillou et al. provides close to the experimental result for 2-layer confinement. For theoretical predictions of Type-2 concrete having 3-layer confinement, the models given by Legeron and Paultre and Triantafillou et al. yield better predictions. Theoretical results of strain models in Table 6 and Table 7 show that only a few of the ultimate strain models are on the lower side, whereas most of the models overestimated the experimental results. Only the strain model by Benzaid et al. is in the range of experimental data for both concrete types and for single, double, as well as three layers of HFRP confinement.

It is evident from the above discussion that existing stress–strain models developed for FRP confinement cannot predict the ultimate strength and strain of HFRP-confined concrete. Therefore, analytical models specifically developed for hemp confinement must be proposed. The following section presents a regression-based approach to predict the compressive stress–strain curves of HFRP-confined concrete.

## 5. Proposed Approach to Predict Compressive Stress–Strain Curves of Hemp-Confined Concrete

### 5.1. Ultimate Compressive Strength $f_{cc}$

**Figure 16:**
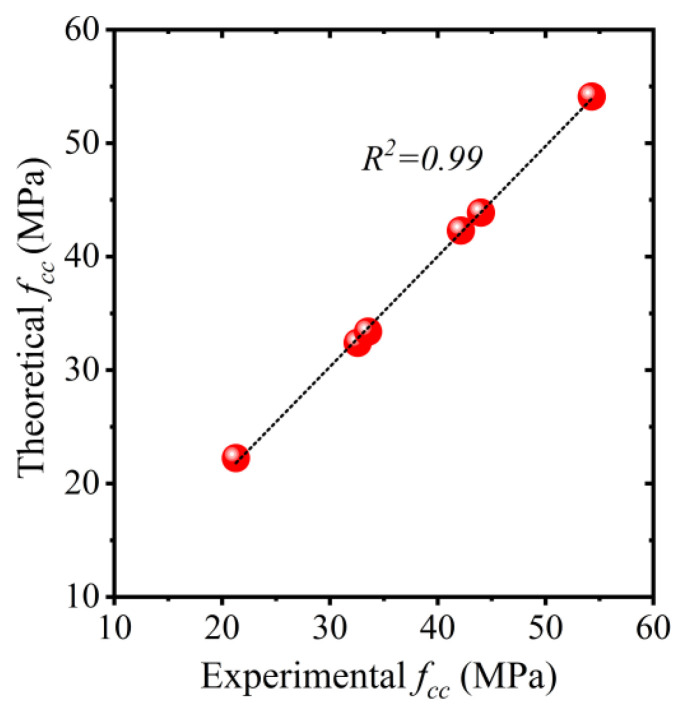
Comparison of experimental vs. predicted compressive strength of hemp-confined concrete.

It is discussed in Section 3 that the compressive strength of HFRP-confined concrete $f_{cc}$ was dependent on the number of external hemp layers and unconfined concrete strength. The effect of the number of hemp layers on compressive strength was determined by estimating the equivalent lateral confining pressure $f_{l}$ using Equation (2). The lateral confinement pressure $f_{l}$ for 1, 2, and 3 layers of hemp confinement was estimated as 3.85 MPa, 7.7 MPa, and 11.55 MPa, respectively. The form of Equation (3) was found to correlate well with experimental data.

(3) $$f_{cc}=\left[1+a{\left(\frac{f_{l}}{f_{co}}\right)}^{b}\right]f_{co}\;$$

where a and b are coefficients of regression. Nonlinear regression analysis was performed to estimate the coefficients a and b. The following equation was obtained from nonlinear regression analysis. The comparison of predicted vs. experimental ultimate compressive strengths $f_{cc}$ is shown in Figure 16. An $R^{2}$ value of 0.99 suggests the efficiency of Equation (4) to accurately predict the ultimate compressive strength of HFRP-confined concrete.

(4) $$f_{cc}=\left[1+2.682{\left(\frac{f_{l}}{f_{co}}\right)}^{0.95}\right]f_{co}\;$$

### 5.2. Ultimate Compressive Strain $\epsilon_{cc}$

**Figure 17:**
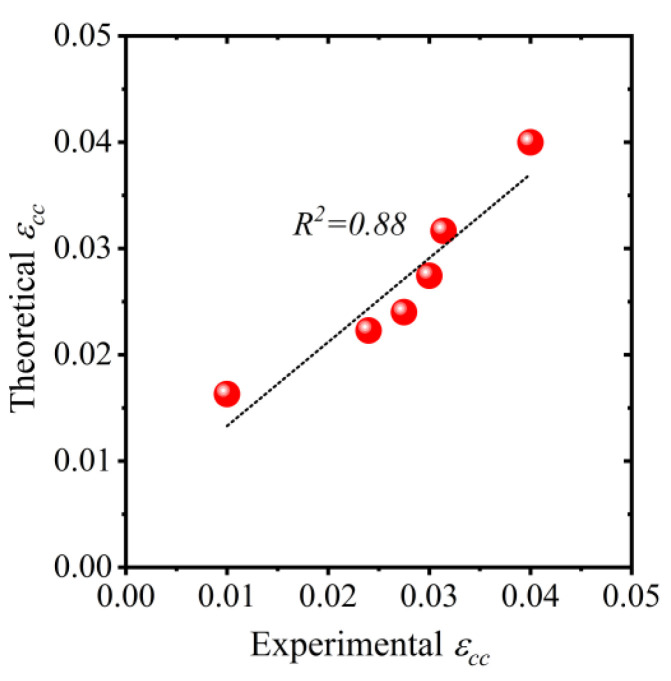
Comparison of experimental vs. predicted ultimate compressive strain of hemp confined concrete.

Existing confinement models were also unable to accurately predict the ultimate compressive strain of HFRP-confined concrete. Therefore, Equation (5) was proposed for the ultimate compressive strain of HFRP-confined concrete. The $R^{2}$ value associated with Equation (5) was 0.88, as shown in Figure 17.

(5) $$\epsilon_{cc}=\left[1+4.650{\left(\frac{f_{l}}{f_{co}}\right)}^{0.717}\right]\epsilon_{co}\;$$

where $\epsilon_{co}$ is the ultimate compressive strain of unconfined concrete fabricated using flaky brick aggregates. A value of 0.007 for $\epsilon_{co}$ was used for regression analysis based on the experimental results reported in the present study.

### 5.3. Prediction of Compressive Stress–Strain Response of HFRP-Confined Concrete

**Figure 18:**
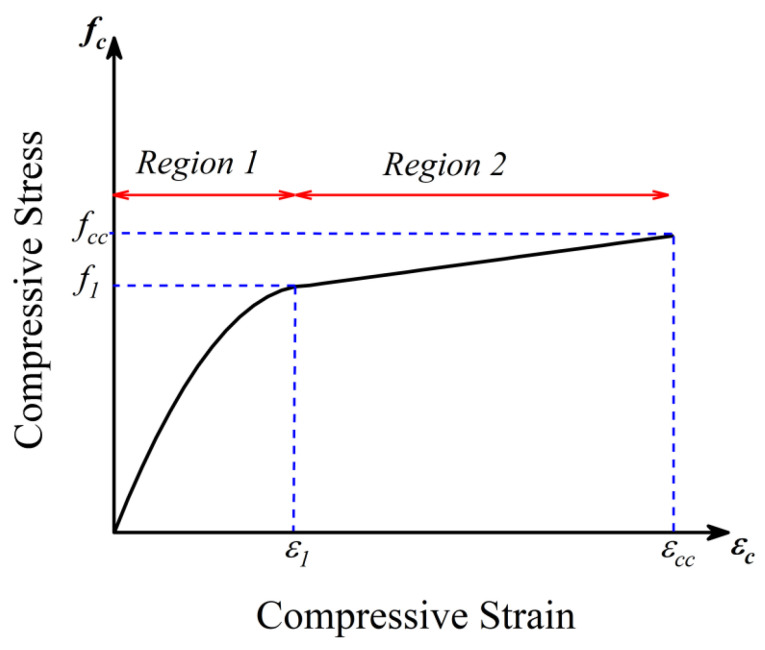
Idealization of compressive stress vs. strain response of hemp-confined concrete.

A two-stage relationship between the compressive stress and strain of HFRP-confined concrete is proposed in the present study, as shown in Figure 18. The stress vs. strain curve is idealized into two regions: (a) Region 1 presents a parabolic ascending branch up to the intersection point corresponding to the compressive stress $f_{1}$; (b) similar to region 1, region two also presents a linear branch up to the ultimate compressive stress $f_{cc}$. However, the elastic modulus of the stress-strain curve of region 2 was much lower than that in region 1. This observed two-stage behavior of hemp-FRP-confined concrete has also been extensively reported for FRP-confined concrete [41].

#### 5.3.1. Compressive Stress vs. Strain Response in Region 1

Scott et al. [56] described the ascending branch of the compressive stress vs. strain response of unconfined concrete or concrete confined with steel ties by using a second-degree parabola as defined in Equation (6). The same form of Equation (6) was used by Harajli [57] to define the ascending branch of the compressive stress vs. strain response of concrete confined by FRP. Thus, Equation (6) was used to define the compressive stress vs. strain response of HFRP-confined concrete. However, Equation (6) was originally proposed for normal coarse aggregate concrete with an α value of 2.0. The current study investigates concrete with recycled brick aggregates. An α value of 1.30 was found appropriate to approximate the initial slope of the compressive stress–strain curve of recycled brick aggregate concrete.

(6) $$f_{c}=f_{1}\left[\frac{2\epsilon_{c}}{\epsilon_{1}}-{\left(\frac{\epsilon_{c}}{\epsilon_{1}}\right)}^{\alpha }\right]\;\;\;\;\;\;\;for\;\epsilon_{c}\leq \epsilon_{1}\;$$

where $f_{1}$ and $\epsilon_{1}$ are the coordinates of the intersection point in Figure 18, and $f_{c}$ is a generic stress value in Region 1 corresponding to the strain $\epsilon_{c}$.

**Figure 19:**
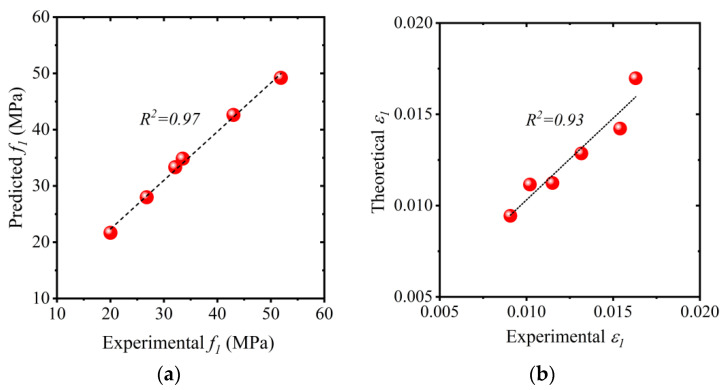
Comparison of experimental vs. predicted (**a**) $f_{1}$ values and (**b**) $\epsilon_{1}$ values.

To define the ascending branch of HFRP-confined concrete, the values of $f_{1}$ and $\epsilon_{1}$ are required. Based upon nonlinear regression analysis, Equations (7) and (8) were proposed to define $f_{1}$ and $\epsilon_{1}$, respectively. The performance of Equations (7) and (8) was assessed in terms of their respective coefficients of determination $R^{2}$. The $R^{2}$ values of Equations (7) and (8) were 0.97 and 0.93, respectively, as shown in Figure 19.

(7) $$f_{1}=f_{co}\left[1+1.914{\left(\frac{f_{l}}{f_{co}}\right)}^{0.687}\right]\;$$

(8) $$\epsilon_{1}=\epsilon_{co}\left[1+1.404{\left(\frac{f_{l}}{f_{co}}\right)}^{0.798}\right]\;$$

#### 5.3.2. Compressive Stress vs. Strain Response in Region 2

The stress vs. strain response in Region 2 is assumed to be linear with a slope $E_{2}$ defined by Equation (9).

(9) $$E_{2}=\frac{f_{cc}-f_{1}}{\epsilon_{cc}-\epsilon_{1}}\;$$

Once parameters $f_{cc}$, $\epsilon_{cc}$, $f_{1}$, and $\epsilon_{1}$ are estimated, the stress vs. strain response in Region 2 can be readily computed.

#### 5.3.3. Comparison of Predicted vs. Experimental Stress–Strain Curves

**Figure 20:**
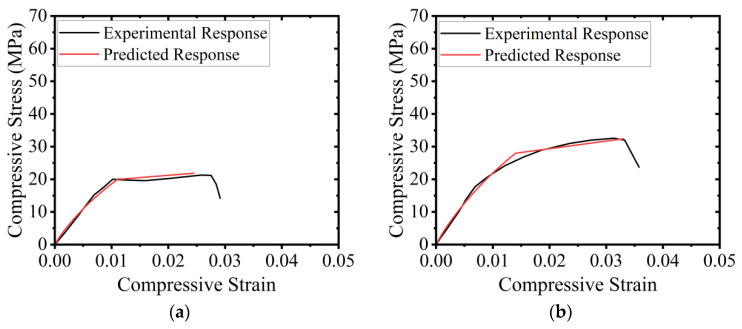

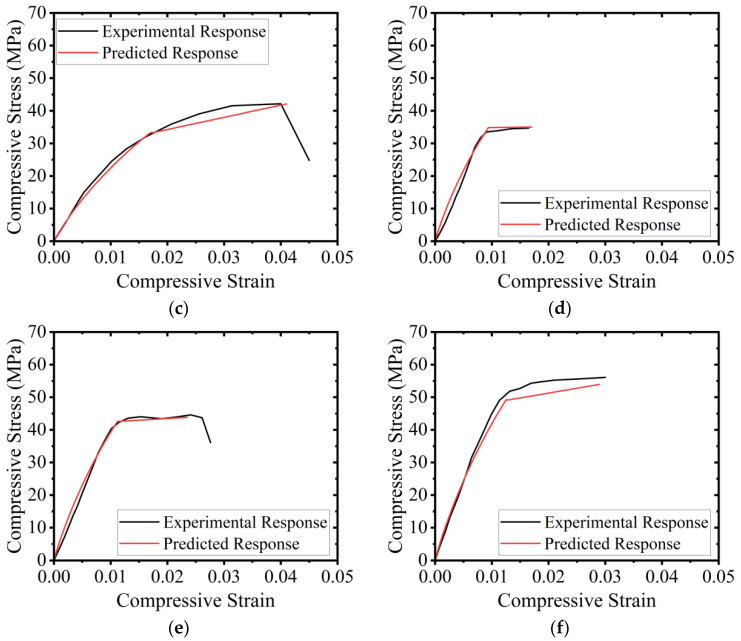
Comparison of experimental vs. predicted compressive stress–strain curves for hemp-confined concrete: (**a**) C-LS-CBB-1H, (**b**) C-LS-CBB-2H, (**c**) C-LS-CBB-3H, (**d**) C-HS-CBB-1H, (**e**) C-HS-CBB-2H, and (**f**) C-HS-CBB-3H.

The approach proposed in Section 5.3.1 and Section 5.3.2 was utilized to predict the compressive stress vs. strain response of HFRP-confined concrete. The predicted response was compared with the experimental response and is presented in Figure 20. It can be observed that the proposed methodology to predict stress–strain curves for HFRP-confined concrete resulted in close agreement with the results.

## 6. Conclusions

The use of waste from fired-clay bricks aggregate to partially replace natural aggregates was the focus of this study. The mechanical characteristics of recycled aggregate concrete made of fired-clay brick aggregate are substandard. Hence, this research investigated the use of natural hemp fiber ropes to mitigate this substandard nature. The following important conclusions can be drawn from this research work:

1. Specimens confined with hemp ropes exhibited a bilinear stress–strain response. Contrary to control specimens, the axial ductility was significantly improved as a result of the hemp fiber rope confinement. The peak axial strength and the corresponding strain in Type-1, as well as Type-2 concrete, increased with the increase in the number of hemp rope layers. Furthermore, the maximum compressive strength and strain were observed in the case of the 3-layer confinement. The improvement in the ultimate compressive strength and strain was more pronounced as the compressive strength of unstrengthened concrete decreased. Improvements of up to 272% and 457% in the ultimate compressive strength and strain were observed due to hemp confinement.
2. The ultimate compressive strength and strain were predicted in this research work using existing analytical stress–strain models. Several models were shown to reasonably predict the compressive strengths. However, none of the models could accurately predict peak strain. Therefore, it was found necessary to propose expressions for predicting the characteristic points on the compressive stress–strain envelope of HFRP-confined RAC.
3. Nonlinear regression analysis was conducted to propose expressions to predict the ultimate compressive strength and strain of HFRP-confined concrete constructed by partial replacement of natural coarse aggregates by recycled fired-clay brick aggregates. In addition, expressions for the compressive stress and strain at the end of the initial stiff branch were also proposed. The proposed expressions resulted in good agreement with the experimental results.
4. An analytical procedure was proposed to predict the stress–strain curves of HFRP-confined concrete constructed by partial replacement of natural coarse aggregates by recycled fired-clay brick aggregates. The compressive stress–strain curves of HFRP-confined RAC were idealized into two branches, i.e., a parabolic branch followed by a linear branch. The proposed regression expressions were utilized to trace the full compressive stress–strain curves of HFRP-confined RAC. A close agreement was found between the experimental and analytically predicted stress-strain curves.
5. The results of this study could be used in multiple areas of civil and structural engineering, especially where there is a need to strengthen and or retrofit existing buildings. In addition, the developed models can be utilized for the design and analysis of reinforced concrete structures.

## Figures and Tables

**Table 1:** Details of test specimens.

Specimen Name	Coarse Aggregate Replacement (%)	Number of Hemp Fiber Rope Layers	Number of Specimens
C-LS-CBB-CON	50%	None	2
C-LS-CBB-1H	50%	1	2
C-LS-CBB-2H	50%	2	2
C-LS-CBB-3H	50%	3	2
C-HS-CBB-CON	50%	None	2
C-HS-CBB-1H	50%	1	2
C-HS-CBB-2H	50%	2	2
C-HS-CBB-3H	50%	3	2

**Table 2:** Concrete design mix.

Mix Materials (kg/m^3^)	Type-1 Concrete	Type-2 Concrete
Cement	242	444
Natural coarse aggregates	605	504
Fine aggregates	726	605
Fired-clay brick aggregates	605	504

**Table 3:** Properties of epoxy.

Property	Value
Tensile Strength (MPa)	50
Elongation (%)	2.5
Flexural Strength (MPa)	75
Curing Time (hours)	6–10

**Table 4:** Result summary of Type-1 concrete specimens.

Specimen Name	Ultimate Strain	Increase in Ultimate Strain (%)	Compressive Strength (MPa)	Increase in Strength (%)
C-LS-CBB-CON	0.0072	-	11.340	-
C-LS-CBB-1H	0.0275	258	21.280	88
C-LS-CBB-2H	0.0314	337	32.580	187
C-LS-CBB-3H	0.0400	457	42.160	272
C-HS-CBB-CON	0.0070	-	22.105	-
C-HS-CBB-1H	0.0100	51	33.535	52
C-HS-CBB-2H	0.0240	262	44.012	99
C-HS-CBB-3H	0.0300	350	54.294	146

**Table 5:** Existing models for compressive stress and strain of FRP confined concrete.

Study	Stress Equation	Strain Equation
Ghernouti and Rabehi [47]	$f_{cc}=\left[1+2.038\left(\frac{fl}{f_{co}}\right)\right]f_{co}$	$\varepsilon_{cc}=\left[1+10.56\left(\frac{fl}{f_{co}}\right)\right]\varepsilon_{co}$
Al-Salloum [48]	$f_{cc}=\left[1+2.312\left(\frac{fl}{f_{co}}\right)\right]f_{co}$	$\varepsilon_{cc}=\left[1+0.024\left(\frac{fl}{f_{co}}\right)\right]\varepsilon_{co}$
Candappa et al. [49]	$f_{cc}=\left[1+5\left(\frac{fl}{f_{co}}\right)\right]f_{co}$	$\varepsilon_{cc}=\left[1+20\left(\frac{fl}{f_{co}}\right)\right]\varepsilon_{co}$
Lam and Teng [50]	$f_{cc}=\left[1+2\left(\frac{fl}{f_{co}}\right)\right]f_{co}$	$\varepsilon_{cc}=\left[2+15\left(\frac{fl}{f_{co}}\right)\right]\varepsilon_{co}$
Lu and Hsu [51]	$f_{cc}=\left[1+4\left(\frac{fl}{f_{co}}\right)\right]f_{co}$	$\varepsilon_{cc}=\left[1+19.21\left(\frac{fl}{f_{co}}\right)\right]\varepsilon_{co}$
Legeron and Paultre [52]	$f_{cc}=\left[1+2.4{\left(\frac{fl}{f_{co}}\right)}^{0.70}\right]f_{co}$	$\varepsilon_{cc}=\left[1+35{\left(\frac{fl}{f_{co}}\right)}^{1.20}\right]\varepsilon_{co}$
Triantafillou et al. [53]	$f_{cc}=\left[1+2.79\left(\frac{fl}{f_{co}}\right)\right]f_{co}$	$\varepsilon_{cc}=\left[\varepsilon_{co}+0.082\left(\frac{fl}{f_{co}}\right)\right]$
Akiyama et al. [54]	$f_{cc}=\left[1+2.4{\left(\frac{fl}{f_{co}}\right)}^{0.647}\right]f_{co}$	$\varepsilon_{cc}=\left[\varepsilon_{co}+0.0766\left(\frac{fl}{f_{co}}\right)\right]$
Benzaid et al. [55]	$f_{cc}=\left[1+2.20\left(\frac{fl}{f_{co}}\right)\right]f_{co}$	$\varepsilon_{cc}=\left[2+7.6\left(\frac{fl}{f_{co}}\right)\right]\varepsilon_{co}$

**Table 6:** Comparison of experimental and analytical results for confinement of Type-1 concrete group.

Study	Specimen	$f_{cc}$ (exp) (MPa)	$f_{cc}$ (Theoretical) (MPa)	$\varepsilon_{cc}$ (exp)	$\varepsilon_{cc}$ (Theoretical)
Ghernouti and Rabehi [47]	C-LS-CBB-1H C-LS-CBB-2H C-LS-CBB-3H	21.28 32.58 42.16	19.18 27.02 34.86	0.0257 0.0314 0.0400	0.0329 0.0587 0.0845
Al-Salloum [48]	C-LS-CBB-1H C-LS-CBB-2H C-LS-CBB-3H	21.28 32.58 42.16	20.23 29.12 38.02	0.0257 0.0314 0.0400	0.0072 0.0073 0.0074
Candappa et al. [49]	C-LS-CBB-1H C-LS-CBB-2H C-LS-CBB-3H	21.28 32.58 42.16	30.57 49.81 69.04	0.0257 0.0314 0.0400	0.0560 0.1049 0.1537
Lam and Teng [50]	C-LS-CBB-1H C-LS-CBB-2H C-LS-CBB-3H	21.28 32.58 42.16	19.03 26.72 34.42	0.0257 0.0314 0.0400	0.0510 0.0876 0.1243
Lu and Hsu [51]	C-LS-CBB-1H C-LS-CBB-2H C-LS-CBB-3H	21.28 32.58 42.16	26.72 42.11 57.50	0.0257 0.0314 0.0400	0.0541 0.1010 0.1479
Legeron and Paultre [52]	C-LS-CBB-1H C-LS-CBB-2H C-LS-CBB-3H	21.28 32.58 42.16	24.11 32.08 38.89	0.0257 0.0314 0.0400	0.0760 0.1654 0.2645
Triantafillou et al. [53]	C-LS-CBB-1H C-LS-CBB-2H C-LS-CBB-3H	21.28 32.58 42.16	22.07 32.80 43.54	0.0257 0.0314 0.0400	0.0350 0.0628 0.0906
Akiyama et al. [54]	C-LS-CBB-1H C-LS-CBB-2H C-LS-CBB-3H	21.28 32.58 42.16	24.86 32.51 38.86	0.0257 0.0314 0.0400	0.0331 0.0591 0.0851
Benzaid et al. [55]	C-LS-CBB-1H C-LS-CBB-2H C-LS-CBB-3H	21.28 32.58 42.16	19.80 28.26 36.73	0.0257 0.0314 0.0400	0.0211 0.0278 0.0346

**Table 7:** Comparison of experimental and analytical results for confinement of Type-2 concrete group.

Study	Specimen	$f_{cc}$ (exp) (MPa)	$f_{cc}$ (Theoretical) (MPa)	$\varepsilon_{cc}$ (exp)	$\varepsilon_{cc}$ (Theoretical)
Ghernouti and Rabehi [47]	C-HS-CBB-1H C-HS-CBB-2H C-HS-CBB-3H	33.535 44.012 54.294	29.94 37.78 45.62	0.010 0.024 0.030	0.019 0.032 0.045
Al-Salloum [48]	C-HS-CBB-1H C-HS-CBB-2H C-HS-CBB-3H	33.535 44.012 54.294	30.99 39.89 48.78	0.010 0.024 0.030	0.006 0.007 0.008
Candappa et al. [49]	C-HS-CBB-1H C-HS-CBB-2H C-HS-CBB-3H	33.535 44.012 54.294	41.34 60.57 79.81	0.010 0.024 0.030	0.031 0.055 0.080
Lam and Teng [50]	C-HS-CBB-1H C-HS-CBB-2H C-HS-CBB-3H	33.535 44.012 54.294	29.79 37.49 45.18	0.010 0.024 0.030	0.032 0.050 0.068
Lu and Hsu [51]	C-HS-CBB-1H C-HS-CBB-2H C-HS-CBB-3H	33.535 44.012 54.294	37.49 52.88 68.27	0.010 0.024 0.030	0.030 0.053 0.077
Legeron and Paultre [52]	C-HS-CBB-1H C-HS-CBB-2H C-HS-CBB-3H	33.535 44.012 54.294	37.70 47.44 55.76	0.010 0.024 0.030	0.037 0.076 0.119
Triantafillou et al. [53]	C-HS-CBB-1H C-HS-CBB-2H C-HS-CBB-3H	33.535 44.012 54.294	32.83 43.57 54.30	0.010 0.024 0.030	0.021 0.035 0.049
Akiyama et al. [54]	C-HS-CBB-1H C-HS-CBB-2H C-HS-CBB-3H	33.535 44.012 54.294	39.22 48.90 56.94	0.010 0.024 0.030	0.020 0.033 0.046
Benzaid et al. [55]	C-HS-CBB-1H C-HS-CBB-2H C-HS-CBB-3H	33.535 44.012 54.294	30.56 39.03 47.49	0.010 0.024 0.030	0.017 0.020 0.024

## Data Availability

The data that support the findings of this study are available on request from the corresponding author.
